# Preliminary study of contrast-enhanced ultrasound in combination with blue dye vs. indocyanine green fluorescence, in combination with blue dye for sentinel lymph node biopsy in breast cancer

**DOI:** 10.1186/s12885-019-6165-4

**Published:** 2019-10-11

**Authors:** Yidong Zhou, Yan Li, Feng Mao, Jing Zhang, Qingli Zhu, Songjie Shen, Yan Lin, Xiaohui Zhang, He Liu, Mengsu Xiao, Yuxin Jiang, Qiang Sun

**Affiliations:** 1 Department of Breast Surgery, Peking Union Medical College Hospital, Peking Union Medical College, Chinese Academy of Medical Sciences, Beijing, 100730 People’s Republic of China; 2 Department of Ultrasound, Peking Union Medical College Hospital, Peking Union Medical College, Chinese Academy of Medical Sciences, Beijing, 100730 People’s Republic of China

**Keywords:** Breast cancer, Sentinel lymph node, Biopsy, Contrast-enhanced ultrasound, Indocyanine green fluorescence

## Abstract

**Background:**

This preliminary study aimed to examine the feasibility of sentinel lymph node biopsy (SLNB) using contrast-enhanced ultrasound (CEUS) vs. indocyanine green fluorescence (ICG), combined with blue dye in patients with breast cancer.

**Methods:**

This was a retrospective study of consecutive female patients with invasive stage I-III (based on pre-operative physical examination and imaging) primary breast cancer at the Peking Union Medical College Hospital between 01/2013 and 01/2015 who underwent preoperative SLNB by ICG + blue dye or CEUS + blue dye. The numbers of detected SLNs, detection rates, and recurrence-free survival (RFS) rates were compared between the two groups.

**Results:**

A total of 443 patients were included. The detection rates of SLNs in the CEUS + blue dye and ICG + blue dye groups were 98.4 and 98.1%, respectively (*P* = 0.814). The average numbers of SLNs detected per patient showed no significant difference between the two groups (3.06 ± 1.33 and 3.12 ± 1.31 in the CEUS + blue dye and ICG + blue dye groups, respectively; *P* = 0.659). After a median follow-up of 46 months, five patients in the CEUS + blue dye group and 15 in the ICG + blue dye group had recurrence. RFS rates showed no significant difference (*P* = 0.55).

**Conclusion:**

This preliminary study suggests that CEUS + blue dye and ICG + blue dye are both feasible for SLN detection in breast cancer.

## Background

Breast cancer is currently the most common malignancy in Chinese women [1, 2]. Recent years have witnessed an increase in the incidence of early breast cancer because of related screening programs, improved women’s breast cancer awareness, and ameliorated imaging technologies. Invasive breast cancer is of particular significance because of its propensity to spread to local lymph nodes and then to other organs/sites. Axillary lymph nodes are the most common sites of regional metastasis, and sentinel lymph node (SLN) biopsy (SLNB) is necessary for tumor staging and prognosis. Axillary lymph node dissection (ALND) allows the sampling of lymph nodes but is associated with significant morbidities such as upper extremity numbness, infection, and lymphedema [3]. SLNB allows the first step of staging, and ALND can be omitted in patients with negative SLNs, reducing the likelihood of complications [3]. SLNs are defined as the initial lymph nodes that drain the breast; thus, their histological condition is considered to represent that of the entire axillary region [4].

The current standard SLNB method involves the injection of a technetium-labeled nanocolloid and blue dye interstitially into the breast, either around the tumor via the periareolar procedure [5]. Use of a radioisotope combined with blue dye is a common method for SLNB, but its shortcomings are not negligible [6]. First, SLNs cannot be detected until many hours have elapsed after radioactive colloid injection, which is a challenge to schedule management. Secondly, patients and healthcare workers may express reluctance to radiation exposure [5]. Thirdly, access to radioisotopes is restricted in some countries. These factors limit the use of SLNB worldwide, especially in hospitals of less developed regions.

In China, using blue dye alone is common in SLNB. Although SLNs are dyed, blue dye cannot indicate their localization prior to skin incision. As a result, the identification rate is not as high as that of the dual method (radiotracer and blue dye) [7, 8]. Therefore, alternative techniques for SLNB are actively sought. Such methods should yield a satisfactory SLN identification rate and avoid the need for radioisotopes.

Therefore, new techniques are being developed for SLNB. Among them, indocyanine green fluorescence (ICG) and contrast-enhanced ultrasound (CEUS) have some advantages [5, 6, 9–21]. Recent studies confirmed that ICG or CEUS alone is feasible and safe for SLNB. However, there are limited data on the benefits of combining ICG or CEUS with blue dye.

Therefore, the aim of the present preliminary study was to examine the effectiveness of SLN identification using CEUS vs. ICG, in combination with blue dye. In addition, we attempted to compare breast cancer recurrence rates between both techniques. The present results provide a proof-of-concept for designing prospective trials.

## Methods

### Ethics statement

This study was approved by the independent ethical committee/institutional review board of Peking Union Medical College Hospital (PUMCH). We obtained permission from PUMCH to collect data from the Breast Surgery Department Database. As this was a retrospective study of anonymized data without any contact with the patients, individual consent was not required. The study was performed in accordance with the relevant guidelines and regulations.

### Patients

A retrospective review of the Breast Surgery Department database of PUMCH was performed. Consecutive female patients aged ≥18 years, with invasive primary breast cancer (stages I-III; based on pre-operative physical examination and imaging), who underwent preoperative SLNB using ICG or CEUS combined with blue dye between January 2013 and January 2015 were included for analysis. Preoperatively, these patients had no clinical (as examined by palpation) or radiological signs of lymph node invasion. Patients who received neoadjuvant systemic therapy (including chemotherapy and endocrine therapy) were excluded, as well as those with bilateral breast cancer or a history of axillary surgery. All eligible patients in the database had complete medical information. No patient was lost to follow-up. Follow-up was censored on January 19, 2018.

### Operative procedures

The choice of the SLNB procedure was based on the surgeon’s experience and preference at the time of surgery. All SLNB procedures were performed by the same team of senior and skilled breast surgeons. Undiluted methylene blue (Bailunsi Co., Tianjin, China, 10 mg/ml) was used for both SLNB procedures.

**Fig. 1:**
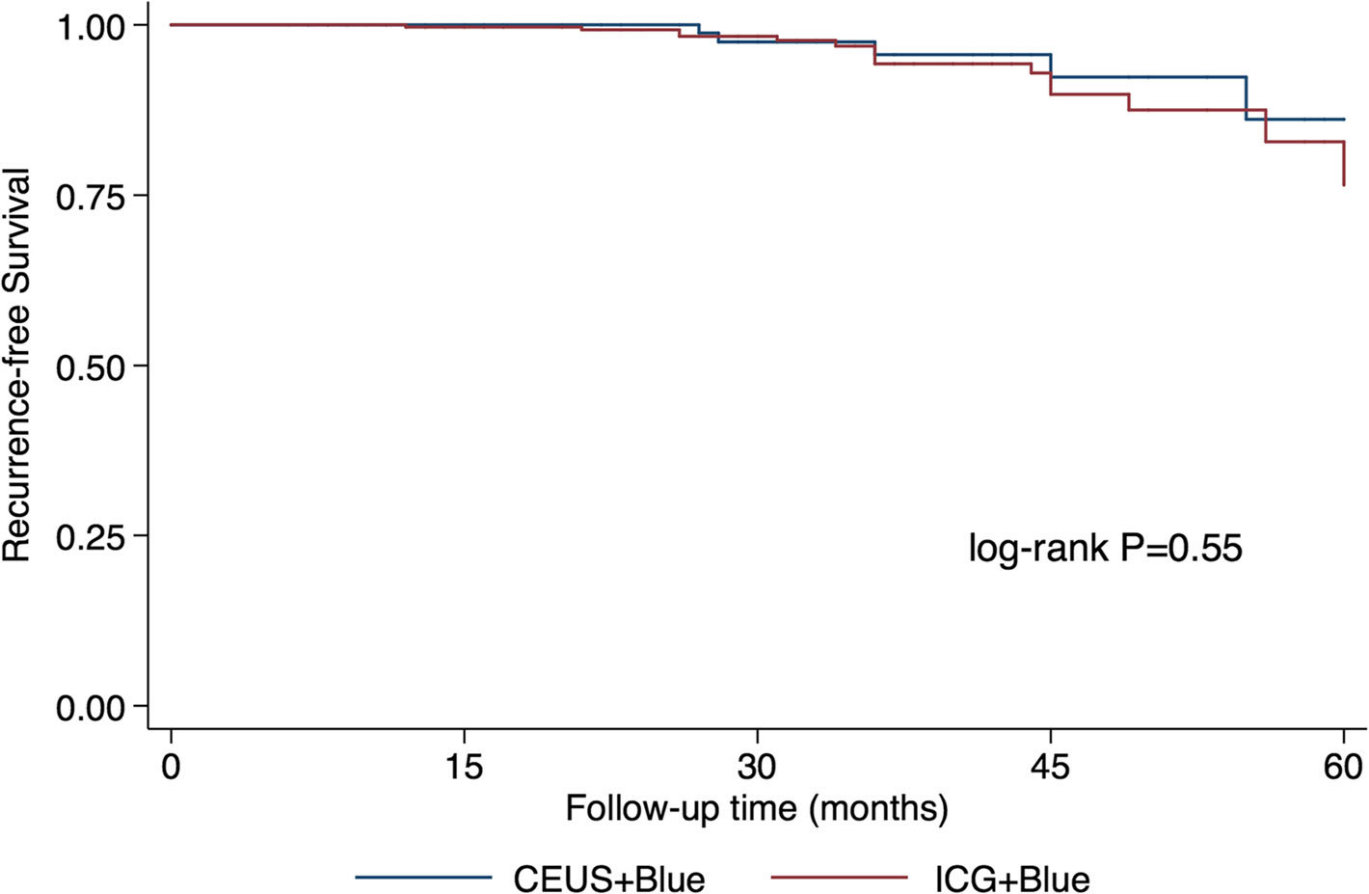
Sentinel lymph node (SLN) localization images. **a** Fluorescent signal mapping of the lymphatic flow and SLNs. **b** SLN detection by the indocyanine green (ICG) + blue dye method. **c** Subcutaneous lymphatic channels and SLNs detected by contrast enhanced ultrasound (CEUS)

For ICG + blue dye, ICG (Dandongyichuang Co., Liaoning, China, 25-mg vial) was first dissolved in 5.0 ml sterile water (5.0 mg/ml stock solution). Then, 1.25 ml of the stock solution was diluted in 5.0 ml sterile water for injection (1.0 mg/ml). Before surgery, 0.2 ml of methylene blue (10 mg/ml) and 0.2 ml of ICG (1.0 mg/ml) were injected intradermally into the periareolar region. The breast was gently massaged for 5 min. Next, the lights were turned off and a photodynamic eye (PDE) camera (Hamamatsu Photonics Co., Hamamatsu, Japan) was used to trace the lymphatic flow. The location of the skin incision for the SLNB was selected as the point where the fluorescent signal disappeared (Fig. 1a). After dissection, the camera was used to confirm the fluorescent signals of SLNs. Blue, fluorescent, and palpable suspicious nodes were all removed and assessed (Fig. 1b).

For CEUS + blue dye, CEUS localization of SLNs was performed in the ultrasound room about 30 min before surgery. Ultrasound was performed on an Acuson S2000 (Siemens Medical Systems, Erlangen, Germany) with 18L6HD and 9 L4 high-frequency linear array probes, using contrast pulse sequences (CPS). Low mechanical index (MI) values were used (MI = 0.06) to reduce cavitation and microbubble destruction. Sonovue (Bracco Imaging, Milan, Italy) was used as the contrast agent. The Sonovue powder was mixed with 2.0 ml of sterile saline. The ultrasound contrast agent (0.4 ml) was injected intradermally into the periareolar area, and the injection area was gently massaged for 10-30 s. Subcutaneous lymphatic channels could be visualized immediately on CPS. Enhanced lymph nodes were detected by moving the probe along the channels (Fig. 1c). Grey scale or live dual images were used to confirm the presence of SLNs. Once identified, lymphatic duct and SLNs were marked on the skin to guide the incision. After CEUS localization, the patients were transferred to the operating room (OR), where the blue dye tracing procedure was performed as described above. Blue, CEUS-localized, and palpable suspicious nodes were removed and assessed.

### Pathological analysis

All the harvested SLNs underwent routine histopathological examination at approximately 2-mm intervals. Immunohistochemistry was performed for the confirmation of suspected metastases. All analyses were performed by the same team of pathologists.

### Data collection

As this was a retrospective study, the patients were grouped according to the SLNB procedure received. Tumor characteristics and demographic information were collected from medical records, including age, menopausal status, tumor size, tumor grade, tumor stage, ER, PR, HER2, and detailed information about SLNB procedures. Follow-up data were reviewed from the hospital’s follow-up system. The adverse events routinely documented after SLNB included lymphedema, infection, sensory deficit, and shoulder function deficit.

### Statistical analysis

The detection rate of SLNs was defined as the number of patients with SLNs identified by the labeling technique divided by the total number of patients administered the technique. Categorical data were compared by the two-tailed chi-square test. Quantitative data were compared by Student’s t-test. Recurrence-free survival (RFS) was estimated by the Kaplan-Meier method. A two-sided log-rank test for time-to-event endpoint was used. Differences were considered statistically significant at *P* < 0.05. Statistical analyses were performed with STATA (version 14.0, StataCorp LP, College Station, TX, USA).

## Results

### Patient and tumor characteristics

Between January 2013 and January 2015, a total of 443 patients were operated and included in this study. The ICG + blue dye technique was used in 316 (71.3%) individuals, and CEUS + blue dye in 127 (28.7%) patients. Table 1 presents the characteristics of both groups. There were no significant differences in age, menopausal status, and breast surgical treatment between the two groups (all *P* > 0.05). There were also no significant differences between the two groups in tumor size, tumor grade, stage, lymphovascular invasion, estrogen receptor (ER), progesterone receptor (PR), and human epidermal growth factor receptor 2 (HER2) (all *P* > 0.05). No adverse reactions or complications related to the ICG procedure, microbubbles, or blue dye injection were recorded.

**Table 1:** Characteristics of the patients

Characteristics	CEUS + blue dye (*n* = 127)	ICG + blue dye (*n* = 316)	*P*
Age, years			0.891
Mean ± SD	45.0 ± 14.5	46.9 ± 15.0	
Menopausal status, *n* (%)			0.987
Premenopausal	72 (56.7)	180 (57.0)	
Postmenopausal	47 (37.0)	115 (36.4)	
Unknown	8 (6.3)	21 (6.6)	
Tumor stage, *n* (%)			0.287
T1	80 (63.0)	217 (68.7)	
T2	40 (31.5)	90 (28.5)	
T3	7 (5.5)	9 (2.8)	
Tumor grade, *n* (%)			0.466
G1	20 (15.7)	54 (17.1)	
G2	64 (50.4)	139 (44.0)	
G3	43 (33.9)	123 (38.9)	
Tumor stage, *n* (%)			0.841
I	74 (58.3)	193 (61.1)	
II	50 (39.4)	117 (37.0)	
III	3 (2.3)	6 (1.9)	
LVI, *n* (%)			0.745
Yes	7 (5.5)	20 (6.3)	
No	120 (94.5)	296 (93.7)	
ER status, *n* (%)			0.682
Positive	97 (76.4)	230 (72.8)	
Negative	23 (18.1)	69 (21.8)	
unknown	7 (5.5)	17 (5.4)	
PR status, *n* (%)			0.768
Positive	91 (71.7)	219 (69.4)	
Negative	29 (22.8)	82 (25.9)	
unknown	7 (5.5)	15 (4.7)	
HER2 status, *n* (%)			0.879
Positive	24 (18.9)	56 (17.7)	
Negative	95 (74.8)	243 (76.9)	
Equivocal /unknown	8 (6.3)	17 (5.4)	
Breast surgery, *n* (%)			0.518
Lumpectomy	50 (39.4)	135 (42.7)	
Mastectomy	77 (60.6)	181 (57.3)	

*CEUS* Contrast-enhanced ultrasound, *ICG* Indocyanine green, *SD* Standard deviation, *LVI* Lymphovascular invasion, *ER* Estrogen receptor, *PR* Progesterone receptor, *HER2* Human epidermal growth factor receptor 2

### Assessment of the two novel dual techniques

Among the 127 patients in the CEUS + blue dye group, SLN detection was successful in 125 (98.4%). Of the 316 patients administered ICG + blue dye, SLN detection was successful in 310 (98.1%). The SLN detection rate showed no significant difference (*P* = 0.814). The numbers of SLNs identified showed no significant difference between the two groups (3.06 ± 1.33 and 3.12 ± 1.31, respectively; *P* = 0.659). There were no significant differences in the positive SLN rate between the two groups. Precisely, there were 13 (10.2%) patients (11 with macrometastases, 1 with micrometastasis, and 1 with isolated tumor cells) with positive SLNs in the CEUS + blue dye group, and 36 (11.4%) (30 with macrometastases, 3 with only micrometastases, and 3 with isolated tumor cells) in the ICG + blue dye group (*P* = 0.726) (Table 2).

**Table 2:** Comparison of sentinel lymph node biopsy results between the two groups

	CEUS + blue dye (*n* = 127)	ICG + blue dye (*n* = 316)	*P*
Identification rate of SLNs, *n* (%)	125/127 (98.4)	310/316 (98.1)	0.814
Number of SLNs identified per patient, mean ± SD	3.06 ± 1.33	3.12 ± 1.31	0.659
SLN metastasis, *n* (%)	13/127 (10.2)	36/316 (11.4)	0.726
Time consumption of SLN localization in the OR (min)	11.01 ± 3.56	12.10 ± 3.21	0.105

*CEUS* Contrast-enhanced ultrasound, *ICG* Indocyanine green, *SLN* Sentinel lymph node, *SD* Standard deviation, *OR* Operating room

The time to SLN localization in the OR showed no significant difference between the two groups (11.01 ± 3.56 vs. 12.10 ± 3.21 min, *P* = 0.105) (Table 2).

All the 49 SLN-positive patients underwent complete ALND, except 1 (isolated tumor cells) in the CEUS + blue dye group, and 5 (including 2 and 3 with micrometastases and isolated tumor cells, respectively) in the ICG + blue dye group.

### Recurrence-free survival

**Fig. 2:**
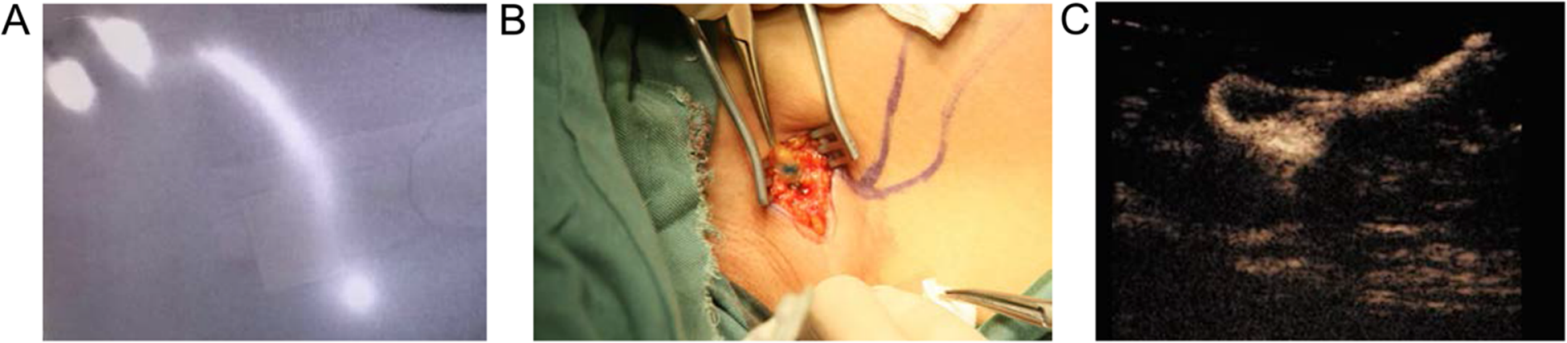
Recurrence-free survival in the contrast enhanced ultrasound (CEUS) + blue dye and indocyanine green (ICG) + blue dye groups

Median follow-up was 46 (range, 8-60) months. Among the 443 patients, 20 (4.5%) had tumor recurrence. Five (3.9%) individuals in the CEUS + blue dye group had recurrence, including 1, 2 and 2 with axillary recurrence, ipsilateral breast/chest wall recurrence and bone metastasis, respectively. A total of 15 (4.7%) patients in the ICG + blue dye group showed recurrence, including 3, 4, 4 and 4 with axillary recurrence, ipsilateral breast/chest wall recurrence, bone metastasis and lung metastasis, respectively. The 3-year RFS was 95.6% in the CEUS + blue dye group versus 94.3% in the ICG + blue dye group (*P* = 0.55) (Fig. 2). No patient died during follow-up.

Regarding cases with axillary recurrence, the patient in the CEUS + blue dye group was a 35-year old woman, whose SLNB showed 0/3 positive SLN. In the ICH + blue dye group, 2 patients (51- and 42-year old women) had 0/3 and 0/4 positive SLN, respectively; the third patient, a 38-year old woman, had 1/4 positive SLN for a macrometastasis and underwent ALND, with 0/18 positive lymph node.

## Discussion

ICG, a novel technique for SLNB, is increasingly used in clinical practice. The SLNB detection rate with ICG alone ranges from 93.1 to 100%, for 1.5-5.4 sentinel lymph nodes sampled per patient [5, 6, 11, 16–18, 20, 21]. The combined use of the conventional blue dye with ICG fluorescence could improve SLN localization and potentially reduce surgical time [22–24]. This combination makes the SLNB procedure easier to perform.

CEUS is another new technique for SLNB and has been validated in a pig melanoma model [25, 26]. Subsequent studies confirmed that CEUS is safe and reliable for SLNB. In 2010, Sever et al. used CEUS for SLNB, and reported a sensitivity of up to 89% [19]. Cox et al. reported a study of 347 breast cancer patients and revealed a detection rate of 87.7% [14]. Esfehani et al. detected lymphatic pathways and SLNs by CEUS alone, with a sensitivity as high as 96% [15]. The CEUS enhancement patterns may help recognize metastatic SLNs and determine the total axillary nodal burden [12, 13]. In addition, CEUS and ICG allow real-time observation of the lymphatic flow in the axilla. Therefore, CEUS can help surgeons plan surgery prior to any incision [13].

In the present preliminary study, the detection rates of SLNs for the two techniques were high and comparable: 98.4% for CEUS + blue dye, and 98.1% for ICG + blue dye. These rates are similar to that (96%) reported in the literature [27, 28]. The two techniques detected > 3 SLNs per patient, without a significant difference. Regarding time consumption in the OR, because the CEUS procedure was performed outside the OR, it is reasonable to expect a shorter localization time in the OR for the CEUS + blue dye technique, implying that the latter method might have a potentially higher efficiency of OR usage. However, no significant difference was observed in the present study between the two methods.

Without performing ALND in all patients, the real false-negative rate could not be determined, but the rate of ipsilateral axillary recurrence could be used as an imperfect adjunct. In the present study, the recurrence rates in the ipsilateral axilla were low [1/127 (0.8%) and 3/316 (1.0%)], suggesting that false-negative rates for both approaches were most likely low. The reported false-negative rate for SLNB is 5-13%, depending upon the number of SLNs sampled, the SLNB method applied, and the cancer type [27, 29, 30]. In the present study, false negative rates based on regional recurrence were lower than previously reported, suggesting a probable underestimation. Among the four patients with axillary recurrence, only one had a positive SLN; she underwent ALND, and all the dissected lymph nodes were negative. Indeed, it is still possible to miss positive lymph nodes during ALND, or the surgeon may decide to not dissect all three levels. In addition, lymph nodes harboring isolated tumor cells may remain clinically negative for a long time and even never develop overt metastasis [31], although conflicting data were reported [32]. Nevertheless, a meta-analysis revealed that dual techniques for SLNB result in lower false negative rates than the use of blue dye alone [28].

In addition to axillary recurrence cases, six (1.4%) patients had ipsilateral breast/chest wall recurrence and 10 (2.3%) developed distant metastasis during the 46-month follow-up. These rates were similar to those reported previously [33–35]. However, such comparison should be interpreted with caution because rates may vary widely when considering the type of breast cancer, the HER2 status, surgical and adjuvant treatments, ethnicity, life style habits, and the follow-up itself.

This study had limitations. ALND was not performed in all patients, and the false-negative rates of the two novel techniques could not be evaluated. Even though there were no significant differences in baseline patient and tumor characteristics between the two groups, a retrospective analysis inevitably has some biases, e.g. we were limited to the data available in medical charts. Furthermore, the surgeons were free to select the preferred method for different patients, and the exact reasons for method selection were usually not indicated in patient charts. Finally, patients assessed by the radiotracer + blue dye technique could not be included because our center does not use radiotracers.

## Conclusion

Overall, the present preliminary study suggested that CEUS + blue dye and ICG + blue dye are both feasible techniques for SLNB in breast cancer. Randomized controlled trials including the radiotracer + blue dye gold standard technique are required to confirm the feasibility, efficacy, and safety of these two novel techniques before their introduction into mainstream clinical practice.

## Data Availability

The raw data are available upon request to the corresponding author and/or to the first author.

## Abbreviations

CEUS: Contrast enhance ultrasound
CPS: Contrast pulse sequences
ER: Estrogen receptor
HER2: Human epidermal growth factor receptor 2
ICG: Indocyanine green fluorescence
MI: Mechanical index
OR: Operating room
PDE: Photodynamic eye
PR: Progesterone receptor
PUMCH: Peking Union Medical College Hospital
RFS: Recurrence-free survival
SLNB: Sentinel lymph nodes biopsy
